# Ground Truth

**DOI:** 10.4056/sigs.50595

**Published:** 2009-09-24

**Authors:** George M. Garrity

**Affiliations:** Department of Microbiology and Molecular Genetics, Michigan State University, East Lansing, Michigan, USA

The concept of ground truth is a well-established principle in cartography, where data collected at a distance are confirmed by measurements made on location. Those local measurements are used to calibrate remote sensing devices, verify or correct experimental inferences, and update geographic databases. Ground truth observations also provide a means of training and supervising image classification software and resolving errors of omission or commission. Cartographic methods have improved significantly because of the development of precise positioning methods (GPS), the development of interoperable data standards for rapid exchange of precise and highly interlinked information, and the development of various devices and visualizations that serve-up information on-demand to different classes of end-users.

There are strong parallels between mapping geographical and biological space. Much of what is underway in genomics, evolutionary biology and systems biology is analogous to the development of a coordinate system onto which living systems can be mapped, natural boundaries and interrelationships uncovered, and predictions of properties and behaviors based. However, it is likely that the dimensionality of any biological coordinate system will exceed the four dimensions of the geographical system. This will confound visualization and complicate “navigation” through biological space, whether it is for purely exploratory purposes or to get from one point to another.

It is a given that the volume of biological data will continue to grow super-linearly for the foreseeable future, as new computational methods are applied to answer the “big questions” in biology. In the absence of major innovation, it is likely that the gap between the cost of data analysis and the cost of data generation will continue to widen. The outcome of such analyses are highly dependent on the quality of the input data, including the underlying information and knowledge used to inform the creation of datasets, the algorithms used in analyses, and the interpretation. Errors of commission and omission appear to be more common in biological data sets than physical data set and the former are more likely to be affected by semantic ambiguity and hidden biases. What is not yet established is which of the labor-intensive curatorial and interpretive tasks can be automated and what metadata that is absent from the public databases may be located and recovered from other sources in a usable form.

The impact of semantic ambiguity in biological data has been noted previously as it pertains to identifiers [1]or biological names [2]. These problems confound accurate and complete retrieval of biological data from public and private databases and from the biological literature; especially in cases in which taxonomic information was misinterpreted or the source organisms were misidentified. Bortolus [3]provides some insights into error cascades in the biological sciences that are attributable to this problem. While his remarks were aimed at field ecologists, the observations apply to computational biologist, modelers, and system biologists as well. So too, do the consequences that such errors have on nature, our knowledge of nature and the socioeconomic costs. Laurin [4] and Hillis [5]provide some additional insight into the potential challenges that will arise as the first adherents of the Phylocode begin to apply their system of nomenclature to plants and animals and their data sets flow into the public repositories. This will add yet another layer of complexity to mining databases and the literature and will represent a source of methodological and theoretical bias that will need to be factored into interpretation of biological data in the future.

This is not an unfamiliar territory to those who have carried out “large-scale” phylogenetic, taxonomic, or ecological analyses in the past. Incorrectly labeled data and data derived from incorrectly identified samples remain common and will continue to confound naive users of public databases and the literature. Tools and techniques to detect and visualize such discrepancies could be useful as components of analytical pipelines, data submissions routines, or as value added service. Authoritatively maintained reference sets of gene and genome sequences derived from taxonomic type material are also needed, especially those that are richly linked to validated phenotypic, physical and geographic metadata and delivered in a highly structured, standards compliant form [6,7]. *The Genomic Encyclopedia of Bacteria and Archaea* [8] represents an outstanding example of an international collaboration that aims to produce such high value data and will provide the ground truth for the next generation of phylogenetic models on which future studies of bacteria and archaea will depend.
